# Methodological strategies to engage young black and Latino heterosexual couples in sexual and reproductive health research

**DOI:** 10.1186/s12913-020-05202-9

**Published:** 2020-05-04

**Authors:** Yzette Lanier, Alena Campo, Claudine Lavarin, Ashley Toussaint, Marya Gwadz, Vincent Guilamo-Ramos

**Affiliations:** 1 New York University, Rory Meyers College of Nursing, New York, NY USA; 2 New York University, College of Global Public Health, New York, NY USA; 3 New York University, Silver School of Social Work, New York, NY USA; 4 Center for Latino Adolescent and Family Health, CLAFH, New York, NY USA

**Keywords:** Implementation science, Couples, Adolescents and young adults, Sexual and reproductive health, Blacks, Latinos

## Abstract

**Background:**

Approaches that move beyond individuals and target couples may be an effective strategy for reducing sexual and reproductive health (SRH) disparities among adolescents and young adults (AYA). However, few researchers have attempted to recruit couples due to feasibility and methodological issues. This study aims to enhance implementation and methodological approaches to successfully engage heterosexual Black and Latino adolescent and young adult (AYA) couples in sexual reproductive health (SRH) research.

**Methods:**

We developed a four-step approach to systematically engage AYA couples in a qualitative study examining factors that influence uptake of combination HIV prevention methods: 1) understanding barriers and facilitators to engaging AYA couples, (2) identifying AYAs living in geographic areas of HIV vulnerability, (3) recruiting and screening AYA couples, and (4) scheduling and completion of the interview session.

**Results:**

Black and Latino youth aged 16 to 24 and their opposite sex romantic were recruited in the South Bronx, New York from September 2017–May 2018. Three hundred and seventy-two men and women completed screening procedures to determine eligibility for the index participant; 125 were eligible and enrolled into the study. Forty-nine nominated partners (NPs) participated in screening procedures and enrolled into the study. A total of 49 couples enrolled into the study; 23 couples completed study activities.

**Conclusions:**

Developing a systematic recruitment plan aided in successfully engaging Black and Latino heterosexual youth. Nevertheless, barriers to study enrollment remained including locating eligible IPs and screening of the NP. Targeting both young men and women was an effective recruitment strategy. Moreover, dyadic strategies that allow for simultaneous interaction with both couple members may be a beneficial strategy to couples’ study enrollment and completion of study activities.

## Background

Despite comprising only 25% of all sexually active individuals, adolescents and young adults (AYAs) aged 15–24 account for nearly half of the 20 million new sexually transmitted infections (STIs) reported annually; approximately 21% of new HIV diagnoses are among youth aged 13–24 [1, 2]. Although there have been steady declines in teen pregnancy, there were nearly 200,000 teen births in 2017 [3]. Among AYAs, there are pronounced sexual and reproductive health (SRH) disparities, with Blacks and Latinos accounting for disproportionate rates of HIV/STIs and teen pregnancy [4]. Therefore, continued public health efforts directed at improving SRH outcomes among AYAs are warranted.

Unlike other health behaviors, such as smoking or physical activity, sexual behavior is an inherently dyadic experience often situated within the context of the couple. Nevertheless, traditional approaches to addressing SRH disparities among AYAs have largely targeted one individual [5, 6]. This individual-focused orientation does not fully account for the dynamic interaction of the couple and neglects the important role that partners play in sexual decision-making including uptake of HIV/STI and pregnancy prevention methods [7–10]. This has resulted in two major gaps in our sexual health promotion efforts. First, an individual approach does not allow for testing partner effects, in which the perspectives of one couple member influence the behaviors of their partner [6, 11]. Therefore, our current knowledge of how individual and couple-level determinants influence AYA sexual behavior is incomplete. Second, although there is an array of individual-level behavioral interventions that have demonstrated effectiveness in promoting SRH, [12–14] they do not address the immediate context of AYA sexual decision making. Thus, observed behavior changes may not be sustained over time because the intervention is directed at one couple member. Therefore, novel approaches that move beyond individuals and target couples as the unit of behavior change are sorely needed.

Although engaging couples in SRH is imperative, this can be quite difficult as there are various logistical, legal, and ethical issues which pose challenges for successful implementation. Engaging couples necessitates that both couple members be interested, eligible, and concurrently available to participate. Subsequently, study enrollment and completion may take more time and effort. Safeguards must also be in place to protect the confidentiality of both couple members. The recruitment and retention of adolescent couples is even more challenging given that these relationships can often be short-lived and also warrant special protection considerations. For example, research involving minors (individuals 17 years and under) requires consideration of parental consent, protection of minor’s privacy regarding sexual activity and romantic relationship involvement, minors’ capacity to consent to research participation, and adolescents’ ability to consent to receipt of SRH services without parental permission [15–17]. SRH research that includes adolescent couples also warrants attention to local laws regarding age of sexual consent and mandated reporting of abuse [18, 19]. In addition to these concerns, some youth living in urban communities may experience socio-economic barriers, such as unreliable communication methods, unstable housing, and familial obligations, which may further hinder study participation and completion [20].

Currently, the public health and implementation science literature on optimal couple-focused recruitment methods is growing [21–26]. However, research outlining specific methodological strategies to engage AYA couples in SRH research is relatively scarce [24, 27, 28]. The current study extends the extant literature by systematically describing the process of engaging young Black and Latino heterosexual couples in a qualitative study exploring decision-making regarding uptake of combination HIV prevention methods (i.e., male and female condoms, HIV and STI testing, and pre-exposure prophylaxis). We focused on heterosexual couples because young women’s risk for negative SRH outcomes such as HIV/STIs is largely contingent on their male partner’s behavior [29]. Moreover, young heterosexual males have been largely neglected in domestic efforts to improve SRH despite their increased vulnerability for poor SRH outcomes [30]. The current study describes the challenges encountered at each phase of the recruitment and enrollment process and offers specific guidance on approaches to address these challenges.

## Methods

### Overview of study and design

#### Targeted sample

Couples in the study were comprised of the index participant (IP) and their nominated partner (NP). The IP was the member of the couple who the research team made initial contact with to assess his/her interest in and eligibility for the study. The NP was the individual that the IP referred as his/her romantic partner and nominated to participate in the study. Study participation was contingent on both the IP and NP meeting distinct eligibility criteria. The eligibility criteria for the IP included the following: 1) English proficiency, 2) self-identify as Black and/or Latino, 3) aged 16 to 24 years, 3) current residence in a geographic area of HIV vulnerability, and 4) in a reciprocated romantic/dating relationship with a person of the opposite sex. Using various behavioral indicators, relationship status was assessed by asking if s/he were in a romantic relationship. Eligibility criteria for the NP included the following: 1) English proficiency, 2) at least 14 years old, and 3) in a reciprocal romantic relationship with the IP. For all minors, there had to be a less than a 4-year age difference between the IP and NP to be congruent with New York state age of consent laws. Minors were required to provide a signed parental permission form based on IRB requirements. The only exclusion criterion for both the IP and NP was being currently married. The IP and NP did not have to be sexually active to participate in the study.

Eligible couples participated in one interview session. All couples provided written informed consent/assent; minors also provided a signed parental permission form. Following the consent procedures, the IP and NP individually completed a brief demographic and sexual behavior survey. A research assistant then provided a brief overview of male condoms, STI screening, HIV testing, and pre-exposure prophylaxis (PrEP), the four HIV prevention methods discussed during the interview session. The IP and NP then met separately with two trained qualitative interviewers for a 60-min, one-on-one individual interview on socio-cognitive and relationship factors influencing use of the four targeted HIV prevention methods. The interviews were conducted in separate, nonadjacent rooms. The IP and NP then met with a third qualitative interviewer and jointly participated in a 60-min dyadic interview on the development and implementation of an AYA-focused, couple-based HIV intervention. Both couple members received $30 cash and an HIV prevention toolkit that contained male and female condoms, lubricant, a pocket-size condom instructional guide, and a community resource guide listing sites offering free/low-cost sexual and mental health services. The study was approved by the (home institution) Institutional Review Board (IRB).

#### Systematic approach for engaging AYA couples

We used a four-step approach to engage AYA couples. The four steps included: understanding barriers and facilitators to engaging AYA couples, identifying AYAs living in geographic areas of HIV vulnerability, recruiting and screening AYA couples, and scheduling and completion of the interview session.

##### Step 1: understanding barriers and facilitators to engaging AYA couples

We searched the literature to inform our recruitment efforts. However, the literature on the recruitment of young Black and Latino couples in SRH research was limited, and the intersection of our target population’s multiple identities presented special recruitment considerations. Therefore, we reviewed descriptive, qualitative, quantitative, and clinical trial studies that addressed the recruitment and retention of couples and Black and Latino youth. We documented multilevel barriers and facilitators to study participation. We also noted special considerations for the inclusion of couples, in general, and adolescent couples, more specifically. This included maintaining the confidentiality of both couple members, determining whether to obtain or waive parental consent, and considering issues regarding local age of sexual consent laws. Based on this literature review, we developed several strategies related to participant engagement and study implementation. These strategies were developed considering the targeted study objectives and timeline, research design, and available resources, such as number of research staff and budget. We developed a community advisory board (CAB) which was comprised of individuals who lived in the targeted communities and/or worked directly with the target population. We also developed a youth advisory board (YAB) which was comprised of individuals who were from the target population. The CAB/YAB were created to provide guidance on the overall development and implementation of the study. We held separate face-to-face meetings with the CAB/YAB where we discussed the barriers and facilitators to study participation identified by the study team. In most instances, the CAB/YAB identified similar challenges to participant engagement and agreed with the developed strategies. As one example, we understood that compensation would be an important motivating factor for youth to participate in the study. Based on our review of the literature which included other qualitative studies, we believed that $30 for each couple member was an appropriate study incentive. Both the CAB and YAB agreed that cash would be preferred over other types of incentives (i.e., gift cards) and that $30 was an acceptable level of compensation based on the time commitment and the study demand. The CAB/YAB also provided important insights that the study team had not considered. For example, when discussing whether to obtain or waive parental consent, the CAB/YAB offered important cultural-contextual considerations including community norms regarding youth participation in research, feasibility of obtaining parental consent, parental acceptability of adolescent dating relationships, youth concerns regarding parents’ knowledge of their romantic relationships, and parents’ English proficiency. This led the study team to revise certain strategies such as modifying language on study flyers (e.g., changing “romantic relationship” to “dating relationship” due to concerns that parents and youth may interpret romantic relationship to mean a sexual relationship) as well as to develop new strategies such as ensuring that all parental forms (i.e., parental permission form, parental FAQ) were in both English and Spanish. The CAB/YAB were re-engaged as different issues arose during the recruitment and enrollment process. Tables 1 and 2 present implementation strategies that were developed based on input from the study team and the CAB/YAB.

**Table 1:** Implementation strategies for couples-focused sexual and reproductive health research

Implementation Strategies	Steps for Successful Implementation
**Study Development**	
Assemble community and youth advisory boards (C/YAB) and hold meetings to receive guidance on study development and implementation	• Identify key community stakeholders through online searches for youth-serving organizations, community walkthroughs, and/or referrals. • Target individuals who are from the target population, work and/or live in the target communities, have experience working directly with the target population, and/or have a strong background in youth SRH. • Develop C/YAB-specific recruitment materials such as flyers, informational sheets about the study, etc. • Elicit feedback on study development and implementation including optimal recruitment and retention strategies. • Provide incentives (i.e., financial compensation, food/refreshments, etc.) to compensate CAB members for their time and insights. • Develop and/or revise study materials based on feedback.
Pilot test screening methods and study instruments	• Recruit and screen individuals from the targeted communities. • Analyze screening data to assess the feasibility of reaching youth who met the study eligibility criteria. • Facilitate the full study protocol to elicit feedback on youths’ comprehension of study materials (i.e., ambiguous wording, awkward instructional sets, and non-youth-friendly language) and revise accordingly. • Provide constructive feedback to the interview facilitation team (i.e., research assistants, interviewers, etc.) to improve their consent and survey administration procedures and interview techniques.
Develop measures to support participants’ physical and emotional well-being	• Develop a protocol for addressing emotional distress and reports of child/sexual abuse. • Develop a community resource guide containing information on sexual and reproductive and behavioral health services in the targeted communities.
*Special Considerations for Couples*	
Develop measures to protect couples’ privacy and confidentiality	• Obtain a certificate of confidentiality from the funding agency that legally protects the research team from sharing couples’ information with anyone not affiliated with the study team. • Secure research space that has nonadjacent rooms to ensure that couple members’ information remains confidential during concurrent facilitation of the individual interviews. • Hire different qualitative interviewers (when possible) for each interview session to ensure the confidentiality of couples’ information (e.g., one interviewer for male individual interview; one interviewer for female individual interview; one interviewer for dyadic interview).
*Special Considerations for Minors*	
Determine whether to obtain or waive parental consent	• Conduct a thorough review of the literature on medical, legal, and ethical considerations regarding obtaining and waiving parental consent, including minors’ ability to consent to research. • Consult C/YAB on the feasibility of obtaining parental consent and the acceptability of waiving parental consent. • Review state laws on: o minors’ ability to consent to the receipt of sexual and reproductive health services (e.g., HIV/STI testing, contraception). o the legal age at which an individual can consent to participation in sexual activity. o mandatory reporting requirements for statutory rape. • Attend IRB consultation sessions prior to protocol submission to discuss considerations for requesting waiver of parental consent for SRH research. • *If obtaining parental consent*: ensure that all parental materials do not disclose minors’ relationship status and explicitly state that minors’ information would not be shared with parents/guardians. • *If requesting a waiver of parental consent*: provide the context regarding a request for a waiver of parental consent such as protecting minors’ privacy regarding their involvement in dating relationships, laws supporting minors’ ability to consent to their own sexual and reproductive health services (if applicable), and potential implications of requiring parental consent such as decreased interest in study participation.
**Study Implementation**	
Develop a tracking database for eligible participants/couples	• Create an online participant tracking database in data management system (e.g., REDCap). • Keep detailed notes on each participants’ screening and re/scheduling communication, including date, time, name of research staff, contact method(s) used, synopsis of the interaction, and next steps. • Link couples’ information by couple ID number.
Develop a locator form to maintain contact with eligible participants	• Require all eligible participants to complete a locator form, which includes: preferred name, contact information (address, phone number, email address, social media handle, etc.), name and contact information of someone who always knows how to reach the participant, preferred contact methods, best time to contact the participant (day [s] of the week and time of day), and availability to participate in the study activities. • Encourage participants to provide at least three reliable contact methods. Reassure participants that all collected information will remain confidential and not be shared with anyone outside of the research team, including parent(s)/guardians or nominated partner. • Review the locator form with the participants to ensure that the information provided was complete, legible, and accurate. • Collect other important contextual notes (i.e., participants school/work schedules, family obligations, etc.). • Enter contact information in REDCap immediately following each recruitment session.
Create a database for the availability of project staff	• Create a project-specific Google calendar. • Confirm the availability of the research team and research space prior to scheduling interview session with couple. • Send calendar invitations to the interview team following appointment confirmation. Pertinent information includes: o interview time, date, and location. o directions to the interview location. o role/assignments (i.e., individual male/female interviewer, dyadic interviewer, research assistant). o couples’ and interview teams’ contact information.
Secure appropriate resources (i.e., research space, staff, etc.)	• Assess space needs including accessibility to public transportation, operating hours (i.e., day, evening, and weekend availability), protection of couples’ privacy/confidentiality, etc. • Assess staffing needs based on study needs (i.e., participant recruitment, facilitation of the interview session, etc.), protection of couples’ privacy/confidentiality, targeted recruitment goal and timeline, budget. • Consider hiring staff that are: members of the target population (race/ethnicity, age, residence), have prior experience working with the target population and/or in the target communities, background in and strong commitment to sexual and reproductive health issues, and flexible work schedules and availability congruent with the target population (i.e., evenings, weekends). • Hold training sessions with all study staff on proper recruitment/retention and study facilitation procedures. Include role-playing to increase comfort engaging with the target population.

**Table 2:** Individual and couples-focused implementation strategies to engage young couples in SRH research

	Individual-Focused Approaches	Couple-Focused Approaches
**Eligibility & Identification**	• Base couples’ eligibility primarily on the female couple member.	• Base couples’ eligibility on either the female or male couple member.
	• Base couples’ eligibility on the IP. If the IP is not eligible, partner screening is not initiated.	• Base couples’ eligibility on either couple member. If the IP is not eligible, partner screening is still initiated to determine the couples’ eligibility.
	• Identify key recruitment locations where youth congregate.	• Identify recruitment locations where young couples congregate.
	• Identify key recruitment times and dates where youth congregate.	• Identify key recruitment times when young couples are around. Capitalize on important dates where couples may be together (e.g., Valentine’s Day).
**Recruitment & Screening**	• Develop general recruitment flyers that target the population of interest.	• Develop general recruitment flyers that are specific to gender (male or female) and recruitment approach (IP or NP). Ensure that the flyers include language that addresses the values and priorities of the target population. • Provide a detailed study FAQ sheet for IPs to share with the NP, including a study overview, participation requirements, compensation, confidentiality, etc.
	• Target/recruit and screen females. If eligible, the female partner and/or the research team recruits the male partner into the study.	• Target/recruit and screen either females or males. If eligible, the index participant and/or the research team recruits the nominated partner into the study.
	• Target and screen individuals who may be in romantic/dating relationships. If eligible, the IP and/or the research team recruits the NP into the study; the NP calls in for screening at a later time.	• Target and screen couples together on the spot. • Target and screen individuals who may be in romantic/dating relationships. If eligible, the IP contacts their NP on the spot (phone, Facetime, etc.). If the NP is available and interested, partner screening is initiated. If the NP is not available, the IP is coached on how to introduce the study to his/her partner and provided study materials to share with the NP.
**Scheduling & Confirming Appointments**	• Scheduling is facilitated through the index participant.	• For couples that are screened together, scheduling of the interviews should be conducted on the spot. Ensure that the recruitment team has staff/space availability. • For couples that are screened individually, ask the IP to contact their NP to discuss scheduling. If not available, arrange a time to follow up when couples may be together.
	• Direct all follow-up communication (i.e., appointment confirmation, reminders, rescheduling) to the IP.	• Direct all follow-up communication to both couple members using group messaging (e.g., text message, WhatsApp, Google Hangouts, Facebook Messenger, etc.). • Direct follow-up communication to the most responsive couple member.

##### Step 2: identifying AYAs living in geographic areas of HIV vulnerability

We reviewed local surveillance and community health reports to find young Black and Latino men and women at heightened risk for poor SRH outcomes. Among the five NYC boroughs, the Bronx had the highest overall rates of new HIV diagnoses and chlamydia and gonorrhea infections, along with the most cases of heterosexual HIV transmission [31–34]. When examined by age group, the Bronx had the second highest number of new HIV infections among youth aged 13–29, chlamydia and gonorrhea infections among women aged 15–24, and teen births [31]. We then identified specific areas within the Bronx that had the highest rates of HIV/STIs and teen pregnancy and high percentages of residents identifying as Black and/or Latino (at least 90%). A total of four community districts were targeted. Our CAB/YAB, who all worked and/or resided in these communities, noted that improving SRH was a primary community concern.

We then conducted an initial Google search to identify potential recruitment areas in the four targeted communities. Major commercial areas were first identified because of the high levels of foot traffic, which was followed by settings attracting high volumes of youth, such as clinics, community-based organizations, recreation centers, parks, other service settings (e.g., barbershops, beauty salons, restaurants). Search terms included “commercial areas in the South Bronx”, “park and recreational centers in the South Bronx”, and “health clinics in the South Bronx”. We also used terms such as “popular hangout in the South Bronx” and “areas in the South Bronx were adolescents and young adults congregate”. We also looked up settings that were recommended by our CAB/YAB. We then conducted community walkthroughs to become familiar with the overall culture and landscape of each neighborhood. On each walkthrough, we noted the frequency of youth in the area, key locations where youth tended to congregate, high and low peak days/times, and public transportation access. We also located additional sites that were not previously identified during our Internet search. In total, we identified 194 potential recruitment venues.

Next, we created a recruitment map via Google Maps and pinned and labeled the identified recruitment areas not viable for recruitment. The map provided a visual layout of key recruitment areas across the targeted neighborhoods. Prior to recruitment, the study team used the map to guide their recruitment efforts and strategically position themselves in ideal recruitment locations. In the field, the recruitment team used the map to move to other nearby recruitment zones if recruitment was slow. We updated the map as new recruitment areas were identified or as previously identified locations were found to be ineffective.

##### Step 3: recruiting and screening AYA couples

**Fig. 1.**
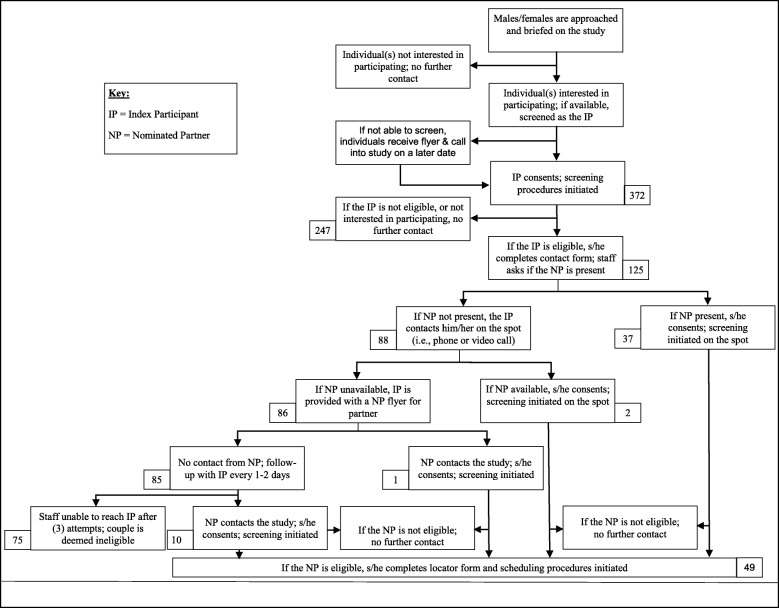
Multi-level approaches for recruiting and screening individuals into the study. ^a^All NPs screened were eligible for the study

Our CAB/YAB indicated that street intercept would be a highly effective strategy for reaching young couples. Therefore, most of our efforts were devoted to this strategy although we also utilized passive recruitment approaches, such as posting study flyers at various venues within the targeted neighborhoods. Figure 1 displays the recruitment and screening process. We approached both men and women to participate in the study based on CAB/YAB feedback. These individuals may have been by themselves or in a group. Most individuals were receptive to hearing about the project. Individuals who expressed an interest in participating in the study were escorted to a quiet area, provided verbal consent for screening, and completed a brief, electronic screening survey. Individuals who expressed interest in study participation but were unable to stop for screening were provided with a flyer containing the basic study information (i.e., study objectives, compensation, contact information, etc.).

**Fig. 2.**
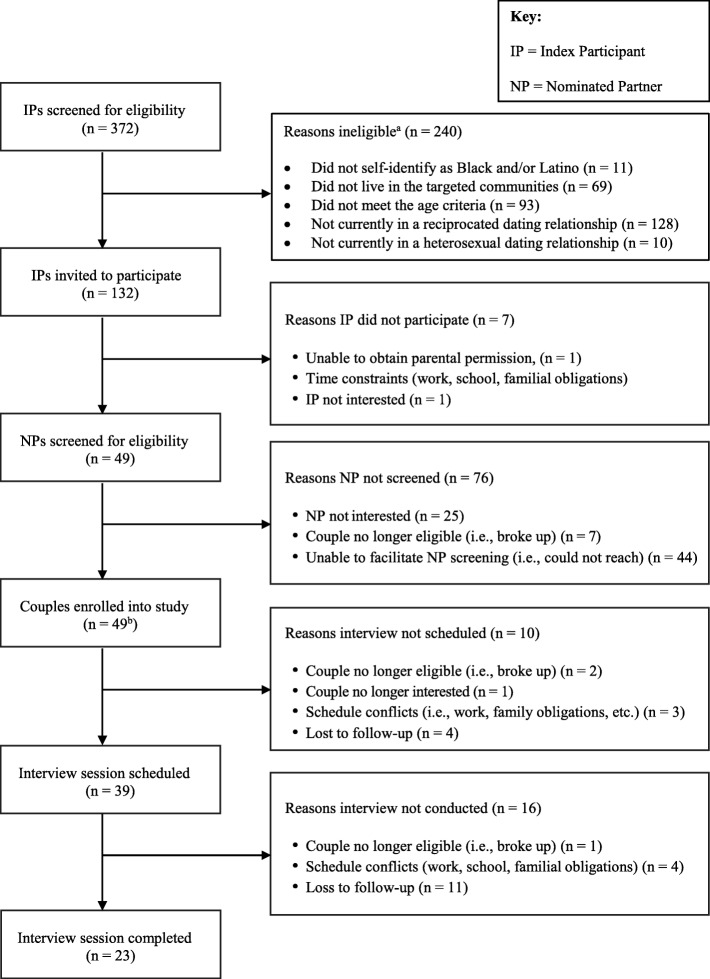
Flow diagram describing engagement of Black and Latino heterosexual couples in the interview session. ^a^ Categories are not mutually exclusive. ^b^ All NPs screened were eligible for and interested in study participation

There were two primary screening approaches. For dyadic screening, we approached a young man or woman (the IP) at random and asked about their interest in the study. If the IP was eligible and their partner (the NP) was present, the NP was also screened. If eligible, both the IP and NP received more detailed study information, including participation requirements and compensation. Interested couples completed a locator form, which included contact information and availability; then, scheduling procedures commenced. Essentially, dyadic screening allowed us to engage with both couple members together on the spot; this was the most ideal and preferred screening scenario. In many instances, however, the NP was not present. In individual screening, once the IP’s eligibility was verified, s/he completed the locator form. We informed the IP that their study participation was contingent on their partner also meeting study eligibility and enrolling. The IP was given the study flyer and encouraged to talk with their partner about the study as soon as possible. We did not collect the NP’s contact information from the IP due to IRB concerns regarding NPs privacy. Therefore, the NP had to contact the study team for screening. For both individual and couple screening, minors were given a parental permission form and a parental FAQ and informed that their participation in the study was also contingent on them presenting the signed document at the interview session. The NP would then contact the study and, if eligible, scheduling would occur. A study cell phone was purchased in order to communicate with potential and enrolled participants via phone calls and text messages. An institutional project email was also set up in order to communicate with potential and enrolled participants who did not have access to a landline or cell phone.

We experienced several challenges that hindered our ability to successfully screen the NP for eligibility. Very few NPs directly contacted the study. We addressed this issue by following up with the IP every 1–2 days via phone, text, and email. However, most IPs did not respond to our emails; they also did not immediately respond to our phone calls or texts. Also, many IPs had disconnected or nonworking telephone numbers. What was particularly challenging about this was that the IPs’ phone service was sporadic (i.e., sometimes phone service would be on and other times off). When we could reach the IP, several expressed no further interest. When offered, reasons for no longer waiting to participate in the study included termination of the relationship and partners being unavailable (i.e., away at school) or not interested in participating. We encouraged individuals to contact the study should they change their minds or if there was a change in their situation. Nevertheless, these individuals were removed from our list of potentially eligible participants and no longer contacted. We learned, however, that the vast majority of IPs had not spoken to their partners about the study but were still interested in participating. In these instances, we asked if their partner was available for screening. If available, the NP was immediately screened. If not, we encouraged the IP to talk to the NP about the study. This process continued until either the NP was screened or contact with the IP was lost.

Our challenges with screening the NP led us to modify both our recruitment and screening methods. First, we began trying to locate couples to increase the number of participants enrolled through dyadic screening methods. As such, we began targeting locations often frequented by young couples. We also implemented another dyadic screening approach. During screening procedures, we asked eligible IPs if they would be comfortable contacting their partners (i.e., phone, Facetime, etc.) on the spot and telling them about the study. If the NP was available and interested, we immediately performed screening. IPs whose partners were not available or decided to forgo this option were asked to provide a time that s/he would either be with their partner or would be available for a follow-up. Once a time had been determined, we provided IPs with a screening appointment reminder card that contained the date and time that a study team member would follow up. To ensure that we were maximizing our time and resources, we also modified our protocol so that individuals were no longer deemed eligible for study participation if there was no contact after three consecutive contact attempts.

##### Step 4: scheduling and completion of the interview session

Following the screening and enrollment of the NP, we proposed potential interview times based on staff and space availability, which were collected bi-weekly and saved into a shared electronic study team calendar. For couples screened through the individual approach, the scheduling procedures were initiated with the NP over the phone. However, most NPs noted needing to consult with their partners before scheduling an interview time. We encouraged the NP to contact the study team once the couple had agreed upon their availability. For couples screened through dyadic approaches, scheduling procedures usually occurred on the spot with both couple members. During dyadic scheduling, couples could talk over their schedules with the recruitment team. We also discussed the time commitment that participation required. Together, this helped couples choose an interview time that best suited their schedules. Couples that expressed needing additional time to determine their schedules were encouraged to immediately follow up with the study.

Generally, we found that couples rarely followed up with the study team. Moreover, couples often did not communicate with each other to coordinate their schedules. This led to significant back and forth between individual couple members and the research team. Therefore, we implemented a new dyadic strategy for couples screened through the individual approach by asking the NP if her/his partner was available. If available, we initiated scheduling procedures with both couple members over the phone. We also began following up with couples 1 day after the initial scheduling attempt. We used group text messaging, which allowed both couple members and the research staff to be involved in the scheduling procedures. We also began directing calls to the most responsive couple member. Similar to screening procedures, the partner served as a broker between the research staff and the couple.

Once couples had been scheduled, we sent an appointment confirmation via text; email confirmation was sent by request. Appointment reminders were sent to both couple members 7 days prior, 1 day prior, and the day of their scheduled appointment. Couples noted that they liked the appointment reminders. Yet many either cancelled or missed/no showed their appointments; some cancelled or missed their scheduled appointments multiple times. Couples who did not show up for their scheduled interviews were immediately contacted for rescheduling. Personal and logistical reasons were the main reasons for missing or cancelling the interview session.

Preparing for and carrying out each interview session required significant time, effort, and resources, including ensuring that there were sufficient staff (e.g., research assistants, interviewers). Therefore, we implemented several strategies to address this issue. First, we modified our protocol so that three missed or cancelled interviews resulted in the couple being removed from the study. Second, we noted that couples’ schedules frequently changed between the 7-day and 1-day reminder. Therefore, the 7-day reminder was replaced with a 3-day reminder. The 3-day reminder allowed us to check-in with the couple and see if there were any circumstances and/or pending obligations that could potentially interfere with their ability to attend the interview session. Based on this information, we could propose an alternative interview time. Third, we implemented an appointment waitlist. In the event a couple cancelled their interview in a timely manner, we contacted other scheduled couples to see if they would be interested in moving up their appointment. Fourth, we attempted to accommodate participants’ schedules in several different ways including offering a grace period which gave couples a window to attend their interview session if they were running late, holding interview sessions in the target community to reduce commute time, and offering roundtrip subway cards to address potential financial challenges.

## Results

As displayed in Fig. 2, 372 individuals were screened for eligibility as the IP. Of these, slightly more than half (52%) were female. One hundred and thirty-two IPs (35%) met the study eligibility criteria and were invited to participate. Of the eligible IPs, 125 enrolled into the study. A total of 49 NPs (which were referred by the IP) completed screening procedures; all were eligible and enrolled in the study. Thus, 49 couples enrolled into the study. Among enrolled couples, 39 (79.6%) scheduled an interview appointment. Twenty-three couples (58.9%) completed the interview session.

## Discussion

Our study highlights several important themes regarding engaging young heterosexual Black and Latino couples in SRH research. First, Black and Latino youth were receptive to participating in couples-based SRH research. There was overwhelming interest in and enthusiasm for the study. Moreover, youth were willing to participate in the study with their romantic partners. The relational aspect of couples-based studies is an important factor in youths’ decisions to engage in research [35]. Hence, recruitment methods should emphasize the interpersonal nature of couples-based studies. Second, although Black and Latino youth are amenable to SRH research, the successful engagement of young couples necessitates the implementation of dyadic approaches. Dyadic methods target the couple as the unit of analysis; this is a radical departure from traditional research methods that typically utilize individual-level approaches. The implementation of dyadic approaches may be especially important for young couples who may experience challenges coordinating and managing their schedules. This warrants a paradigm shift in our recruitment and retention approaches from individual methods to the application of dyadic methods. Third, it is feasible to engage both males and females. Previous couples-based recruitment methods have typically focused on the female partner [21, 23, 24]. However, in the current study, males and females were both approached and equally receptive to study participation. Young Black and Latino heterosexual males have often been characterized as “hard to reach” and largely left out of SRH research despite experiencing a disproportionate burden of sexual health outcomes [36]. Therefore, dyadic methods may be a promising strategy for increased involvement in SRH research among heterosexual males.

There are important caveats that should be considered. Although engaging young couples in SRH research is both acceptable and feasible, it is time- and resource-intensive, taking considerable planning and effort to successfully identify and enroll young couples that meet the eligibility criteria and to facilitate the completion of the study activities. Dyadic methodologies require additional staff and space, which makes the coordination and scheduling of study activities more complex. Moreover, the inclusion of adolescent couples also necessitates special considerations and safeguards that ensure the protection of minors. A needs assessment should be conducted early during study planning to ensure that necessary resources needed to successfully conduct the research are available. This should be considered alongside factors such as the research design, the target population, study timeline, and budget. Despite high effort, couples-based studies often result in a low yield. Consistent with other couple-based studies, there was considerable attrition from initial screening to the completion of study activities. While our enrollment rates were slightly lower than other couple-based survey and intervention studies [27], they were fairly consistent with other dyadic, qualitative studies [37]. Nevertheless, our findings suggest that more couples will need to be approached and screened in order to meet study enrollment goals.

## Conclusion

Development of a systematic recruitment approach aided in addressing challenges associated with engaging young Black and Latino couples in SRH. More research is needed to understand which strategies are most effective in locating, recruiting, and retaining young couples.

## Data Availability

The datasets generated and/or analyzed during the current study are not publicly available due to ongoing analyses.

## Abbreviations

AYA: Adolescents and young adults
CAB: Community advisory board
HIV: Human immunodeficiency virus
IP: Index participant
IRB: Institutional Review Board
NP: Nominated partner
NYC: New York City
PrEP: Pre-exposure prophylaxis
SRH: Sexual and reproductive health
STI: Sexually transmitted infection
YAB: Youth advisory board
